# Applications of New Rhizobacteria *Pseudomonas* Isolates in Agroecology via Fundamental Processes Complementing Plant Growth

**DOI:** 10.1038/s41598-019-49216-8

**Published:** 2019-09-06

**Authors:** R. Qessaoui, R. Bouharroud, J. N. Furze, M. El Aalaoui, H. Akroud, A. Amarraque, J. Van Vaerenbergh, R. Tahzima, E. H. Mayad, B. Chebli

**Affiliations:** 1 Research Unit of Integrated Crop Production, Centre Regional de la Recherche Agronomique d’Agadir, Agadir, Morocco; 2 Biotechnology and Environmental Engineering Team, Laboratory of Mechanic Process Energy and Environment, National School of Applied Sciences, Ibn Zohr University, Agadir, Morocco; 3 Laboratory of Biotechnologies and Valorization of Natural Resources Faculty of Sciences - Agadir, Ibn Zohr University, Agadir, Morocco; 4 Plant Science Unit – Flanders Research Institute for Agriculture, Fisheries and Food (ILVO), Merelbeke, Belgium

**Keywords:** Biotechnology, Ecology, Ecology, Environmental sciences

## Abstract

*Pseudomonas* isolates have frequently been isolated from the rhizosphere of plants, and several of them have been reported as plant growth-promoting rhizobacteria. In the present work, tomato (*Solanum lycopersicum*) seeds were germinated in greenhouse conditions, and the seedling height, length of plants, collar diameter and number of leaves were measured from plants grown in soil inoculated by bacterial isolates. *Pseudomonas* isolates were isolated from the rhizosphere. We used the Newman-Keuls test to ascertain pairwise differences. Isolates were identified as a new *Pseudomonas* species by *rpo*D gene sequencing. The results showed that isolates of *Pseudomonas* sp. (Q6B) increased seed germination (P = 0.01); *Pseudomonas* sp. (Q6B, Q14B, Q7B, Q1B and Q13B) also promoted seedling height (P = 0.01). All five isolates promoted plant length and enlarged the collar diameter (P = 0.01). *Pseudomonas* sp. (Q1B) also increased leaf number (P = 0.01). The investigation found that *Pseudomonas* isolates were able to solubilize phosphate, produce siderophores, ammonia, and indole-3-acetic acid and colonize the roots of tomato plants. This study shows that these five novel *Pseudomonas* sp. isolates can be effective new plant growth-promoting rhizobacteria.

## Introduction

Plant growth-promoting rhizobacteria (PGPR)^1^ are an indispensable part of the rhizospheric biota and are beneficial bacteria that colonize plant roots^2,3^. PGPR increase the growth, yield, and stress tolerance of crop plants^4^. They improve plant growth through enhanced nutrient uptake from soil^5^ and a wide variety of mechanisms such as phosphate solubilization, siderophore production, biological nitrogen fixation, phytohormone production, antifungal activity, and systemic resistance induction. Such mechanisms make PGPR potentially usable biofertilizers^6^. Their emergence as a potent alternative has come in response to the overuse of agrochemical products such as fertilizers and pesticides, which lead to contamination of soil, fruits and vegetables^5^. This threat has prompted field workers to seek viable alternatives to reduce the use of chemical products.

According to studies conducted on this issue, PGPR can be included in biofertilizers and biopesticide applications, which makes them the most effective organic alternative^6–8^. PGPR comprise 2–5% of the total rhizobacterial community^1^ and facilitate its application. In line with the efficacy of PGPR, additional qualities also aid in increasing seed germination^9^, seedling vigor and plant growth^10^. PGPR are beneficial microorganisms that may be used in place of synthetic chemicals; furthermore, they improve plant growth by increasing nutrient availability. Overall, PGPR help to sustain environmental health and soil productivity^11–13^. In the analysis of PGPR, many studies confirm that the *Pseudomonas* genus represents the core of PGPR for many crops^1,14–19^.

Studies detail a twofold action in PGPR function. Direct mechanisms operate to produce metabolites. Kloepper *et al*.^20^ reported the production of metabolites, such as siderophores, by PGPR, contributing to enhanced growth. PGPR are also characterized by their ability to produce plant hormones, such as indole-3-acetic acid (I-3-AA)^21–23^, gibberellic acid^24^ and cytokinins^25^. Furthermore, PGPR solubilize phosphate^26–28^. Mishra *et al*.^29^ described *Pseudomonas* sp. MA-4 as the most efficient producer of ammonia that significantly increased biomass of the medicinal plant *Geranium*. Indirectly, PGPR promote plant growth by colonizing plant roots^30,31^ and suppressing diseases caused by pathogens^32^. Weller^33^ himself found that specific strains have the capacity to colonize the whole root system and survive for several weeks in the presence of the natural microflora. The combination of processes that PGPR contribute to make them an alternative to synthetic chemicals.

Our objective was to investigate the growth-promoting effects of laboratory fluorescent *Pseudomonas* isolates on tomato growth. Experiments were carried out to precisely evaluate the capacity to produce compounds such as those involved in regulation (I-3-AA), growth promotion (siderophore) and nutrient uptake assistance (phosphate solubilization). We also investigated their direct effect on plant height, collar diameter and leaf number in greenhouse conditions.

## Results

### Isolation of *Pseudomonas* sp

As already described above, bacterial populations were quantified from the samples collected from the tomato greenhouse of an experimental farm. The results obtained (Supplement Material Fig. S1) were mainly based on the method of Amkraz *et al*.^34^. They showed that the rhizospheric soil of tomatoes is rich in bacteria. The detected bacterial flora was superior to fluorescent *Pseudomonas* for all samples of roots. The fluorescent *Pseudomonas* were more abundant in the rhizoplane than the rhizosphere or endorhizosphere. They were characterized by discrete classes of 4.1 × 10^5^, 2.9 × 10^5^and 4 × 10^4^ cfu in rhizoplane, rhizospheric soil and endorhizosphere, respectively. They represented 4.24%of the bacteria in the rhizoplane. In our investigation of the 19 fluorescent *Pseudomonas* isolates from all samples, 11 isolates (Q029B, Q052B, Q063B, Q021B, Q13B, Q056B, Q066B, Q059B, Q017B, Q034B and Q14B) were isolated from the rhizoplane, 5 isolates (Q6B, Q003B, Q054B, Q1B and Q7B) from the endorhizosphere and 3 isolates (Q015B, Q007B and Q027B) from the rhizospheric soil.

### Greenhouse assay measuring seed germination and seedling height

**Figure 1:**
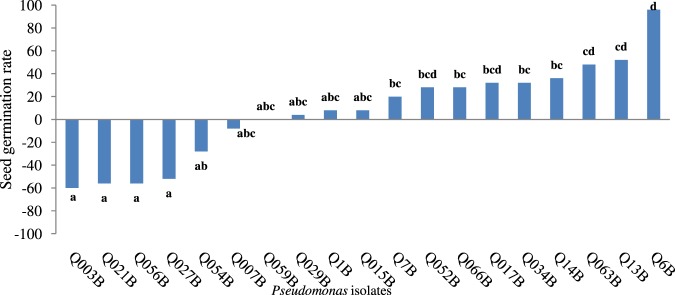
Effect of fluorescent *Pseudomonas* on seed germination compared to that of the control. Bars with the same letters are not significantly different at P < 0.01 using the Newman-Keuls test.

**Figure 2:**
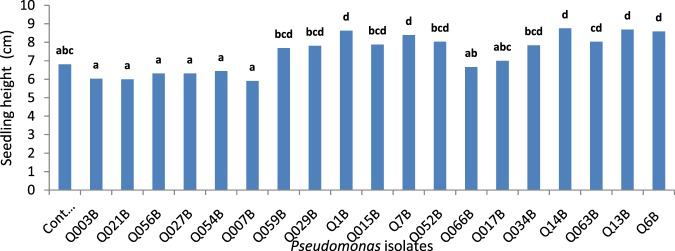
Effect of fluorescent *Pseudomonas* on tomato seedling growth. Bars with the same letters are not significantly different using the Newman-Keuls test (P < 0.01).

Tomato seeds bacterized with fluorescent *Pseudomonas* were distinguished by their potential efficacious effects on seed germination and plant growth. Among the 19 isolates tested, Q6B was characterized by the highest significant effect on germination percentage (96%) compared to that of the control (Fig. 1). Twenty days after germination, the seedling height was enhanced significantly by five *Pseudomonas* isolates (Q14B, Q13B, Q1B, Q6B and Q7B) ranging from 8 cm to 8.75 cm for and Q7B and Q14B, respectively (Fig. 2). Due to such efficacious contributions to seedling height for Q14B, Q13B, Q1B, Q6B and Q7B, it was reasonable to test their effects on plant growth after transplantation.

### Characterization of five fluorescent *Pseudomonas* isolates

**Figure 3:**
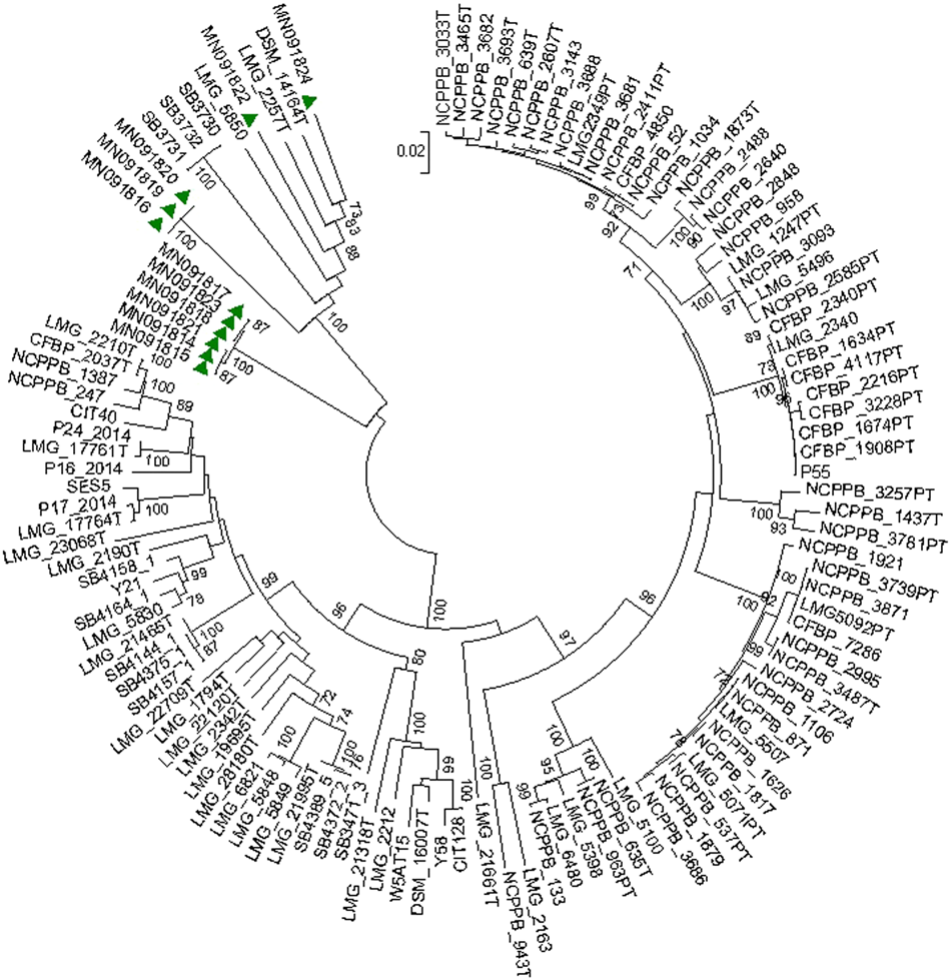
Evolutionary tree representing Maximum likelihood phylogenetic analysis inferred from partial *rpo*D nucleotide sequences of the Moroccan (green triangles) and representative *Pseudomonas* strains. The GenBank accession numbers of the sequences are indicated together with the strain name. BioNumerics analysis included most of the available *rpo*D sequences. The phylogenetic clusters are delineated with vertical bars. Branch lengths on the phylogenetic tree represent the genetic distance, the numbers at the branches represent the percentage of replicates in which the topology of the branch was observed after 500 bootstrap replicates.

The five isolates (Q14B, Q13B, Q1B, Q6B and Q7B) showed fluorescence production with diffusible yellowish-green pigment in KB medium under ultraviolet light (360 nm). The tested isolates were positive for motility and oxidase and arginine dehydrogenase activity. They were gram-negative. The isolates Q6B, Q13B and Q1B were positive for catalase activity and levan production, whereas Q7B and Q14B were negative (Table 1). Q7B and Q1B were positive for nitrate reduction, while Q6B, Q13B and Q14B were negative. Q13B was positive for gelatin liquefaction, but the others were negative (Table 1). Pure cultures of each isolate were used to confirm their identity on a species level using a molecular approach. To identify the isolated bacteria, a partial *rpo*D gene sequence of each isolate was amplified and sequenced. The obtained sequence was submitted to GenBank (MN091814 to MN091824, supplement material Table S1) and used for searching against the GenBank database using BLAST (http://www.blast.ncbi.nlm.nih.gov). The BLAST analysis results showed alignment of the sequence with several species of *Pseudomonas*, where the sequence exhibited maximum identity with *Pseudomonas* sp. Therefore, to characterize each isolate more precisely, the most closely related sequences were used to reconstruct a phylogenetic tree using maximum likelihood analysis. For the Moroccan *Pseudomonas* isolates, maximum likelihood phylogenetic trees inferred from the aligned partial *rpo*D nucleotide sequences from our study and from NCBI provided key information about their relationship within *Pseudomonas* spp. Diverse group. We could reconstruct the entire *Pseudomonas* phylogenetic landscape with all *Pseudomonas* Moroccan isolates grouped into two genetically close clusters. One cluster containing sequences of 5 Moroccan isolates closely related (98% nt similarity) to GenBank sequences of *Pseudomonas* type isolates from USA (LMG_2257T, accession number D86020) and France (DSM14164T, accession number FN554488) (Fig. 3). The other set of sequences from six Moroccan isolates clustered separately in a distant homogenous clade (99% nt similarity) sharing 99% identity with Chinese *Pseudomonas* sp. stains (MH758786). The inferred phylogenetic tree confirmed that the isolated bacteria lie in the genus *Pseudomonas*, are related to newly annotated *Pseudomonas* species, and are constituting a genetically independent branch (Fig. 3). Thus, taken all together, these results strongly indicate that the isolated organisms may belong to a new species in the genus *Pseudomonas* that have not yet been described.

**Table 1:** Biochemical characteristics of five *Pseudomonas* isolates.

	Gram	Fl	Ox	Mt	Ca	N	Arg	L	Gl	Glu*	Suc*	Man*
Q6B	−	+	+	+	+	−	+	+	−	ox	−	−
Q13B	−	+	+	+	+	−	+	+	+	ox	−	−
Q7B	−	+	+	+	−	+	+	−	−	ox/fr	+	+
Q14B	−	+	+	+	−	−	+	−	−	ox/fr	−	−
Q1B	−	+	+	+	+	+	+	+	−	ox/fr	+	+

Fl = fluorescence, Ox = oxidase, Ca = catalase, Mt = motility, N = nitrate, Arg = arginine, L = levan, Gl = gelatin, carbon source (Glu = glucose, Suc = sucrose, Man = mannitol). *Glucose: ox: oxidation, fr: fermentation.

### Plant growth

The performance of *Pseudomonas* isolates on tomato plant growth parameters was observed under greenhouse conditions 20 days after transplantation. The number of leaves, plant length (aerial region) and collar diameter were recorded. The results of this study showed that all of the bacteria significantly promote (p < 0.01) plant length and collar diameter. The longest significant plant length was obtained with Q13B, with a 31.5% increase in plant length (statistical supplementary file) and a 48.57% increase in collar diameter compared to those of the control. *Pseudomonas* sp. (Q1B) significantly enhanced leaf number by 83.33% (P < 0. 01) compared to that of the control (Table 2).

**Table 2:** Influence of fluorescent *Pseudomonas* strains on the number of leaves, plant length and collar diameter.

	PL (cm)	Gain (%)	CD (mm)	Gain (%)	NL	Gain (%)
Control	30.80 ± 2.79^b^	−	5.25 ± 0.67^b^	−	3.00 ± 0.00^a^	−
Q6B	36.20 ± 1.73^a^	17.53	7.00 ± 0.88^a^	33.33	3.90 ± 0.87^a^	30. 00
Q14B	38.90 ± 1.90^a^	26.30	7.22 ± 1.00^a^	37.62	4.30 ± 1.39^ab^	43.33
Q13B	40.50 ± 3.00^a^	31.50	7.80 ± 1.60^a^	48.57	3.80 ± 0.63^a^	26.66
Q7B	37.05 ± 2.47^a^	20.30	6.65 ± 0.58^a^	26.66	4.20 ± 0.94^ab^	40. 00
Q1B	37.50 ± 2.17^a^	21.75	7.55 ± 1.25^a^	43.91	5.50 ± 1.73^b^	83.33

PL: plant length, CD: collar diameter, NL: number of leaves. Values indicate mean values (±SD); different letters indicate significant differences within a row or column at P < 0.01 according to the Newman-Keuls test.

### PGPR mechanisms

The five *Pseudomonas* isolates (Q14B, Q13B, Q1B, Q6B and Q7B) were tested for PGPR activity as follows: phosphate solubilization ability, I-3-AA production, siderophore production, ammonia production and root colonization ability (Table 3). All these isolates solubilized phosphate in solid NBRIP medium. Phosphate solubilization is manifested in the clear halo around the colony. Solubilization is shown for Q13B (11 to 75 mm halo diameter). In liquid medium, these isolates showed successful phosphate solubilization. The best solubilization was shown by Q6B (3.80 mg/ml). The five isolates produce siderophores in both solid and liquid medium. Production is visualized in the orange halo around the colony. The highest level of production was by Q13B (53.8%). The five isolates produce ammonia, with the best production shown by Q1B (3.55 mol/ml). The production of I-3-AA was detected in the five isolates in both solid and liquid media. The presence of I-3-AA was indicated by red spots on Whatman paper (No. 2) after treatment with Salkowski reagent. As far as liquid medium is concerned, the best production was shown by Q1B (2.26 µg/ml). All five isolates are characterized by their capacity to completely colonize the roots of seedlings.

**Table 3:** PGPR effect of five *Pseudomonas* strains. HD: halo diameter, Q: qualitative (+: yes −: no).

	Phosphate solubilization		I-3-AA		Siderophore		Ammonia (mol/ml)	Root colonization
	HD (mm)	mg/ml	Q	µg/ml	Q	%		
Q6B	8.25 ± 0.96^abc^	3.80 ± 0.99^b^	+	1.23 ± 0.00^b^	+	45.61 ± 1.02^a^	0.97 ± 0.02^b^	+
Q14B	5.00 ± 0.00^a^	1.34 ± 0.13^a^	+	1.04 ± 0.01^a^	+	46.66 ± 0.41^a^	2.63 ± 0.04^c^	+
Q13B	11.75 ± 3.20^bc^	1.10 ± 0.17^a^	+	1.79 ± 0.02^c^	+	53.80 ± 0.94^d^	3.08 ± 0.09^a^	+
Q7B	7.50 ± 0.58^ab^	0.89 ± 0.15^a^	+	2.08 ± 0.02^d^	+	26.24 ± 0.37^c^	3.23 ± 0.12^a^	+
Q1B	11.50 ± 1.29^c^	1.16 ± 0.22^a^	+	2.26 ± 0.00^e^	+	17.48 ± 0.04^b^	3.55 ± 0.14^d^	+

Values indicate mean values (±SD); different letters indicate significant differences within a row or column at P < 0.01 according to the Newman-Keuls test.

## Discussion

We achieved the objective of confirming that the *Pseudomonas* isolates focused on in this study facilitate siderophore, I-3-AA, and ammonia production, phosphate solubilization and the growth of tomatoes in greenhouse conditions.

The results showed that the rhizospheric soil of tomatoes is rich in bacteria. The number of aerobic bacteria was superior to fluorescent *Pseudomonas* for all root samples. These results were similar to those reported by Amkraz *et al*.^34^ but with an apparent difference in terms of the percentage of *Pseudomonas* in each sample of roots. The study performed by Amkraz *et al*.^34^ showed that *Pseudomonas* proportions were 7% in rhizospheric soil, 20% in the rhizoplane and 0.21% in the endorhizospheric component compared to 10%, 4.24% and 9.72%, respectively, in our survey. This disparity is attributed to the soil type, plant age, the season in which the samples were collected and the use of pesticides. Bacterial presence is variable, and the heightened sensitivity/susceptibility of bacteria is due to the heterogeneous distribution of bacteria in the soil in relation to farming practices, the use of chemical products, crop type and soil type^19,35^. Under our conditions, *Pseudomonas* was highly represented in comparison to the cultivable bacteria detected.

This study additionally demonstrated that among 19 isolates, five are effective PGPR isolates; they increased seed germination and plant growth of tomatoes under organic growing conditions. Growth stimulation mechanisms, including the production of phytohormones, phosphate solubilization, ammonia production and colonization of plant roots, are the most efficacious mechanisms that explain PGPR effects^35,36^. Chin-A-Woeng *et al*.^37^ reported that the ability of *Pseudomonas* isolates to suppress disease relies mainly on their ability to colonize roots. The results revealed a significant increase in seed germination due to the mixed bacteria formulation compared to the control. This effect is due to the increased synthesis of hormones linked to growth such as I-3-AA and gibberellins, which triggered the activity of specific enzymes that promote early germination^38^.

The molecular characterization showed that the five selected bacteria lie in the genus *Pseudomonas*, and they are a new strain of *Pseudomonas* species that has not yet been described. Phylogenetic relationship of the Moroccan isolates was revealed based on polymorphism in *rpo*D gene region, used earlier as an ecological marker^39^. The sequence of *Pseudomonas*-specific single-copy gene *rpo*D gene from 11 fluorescent pseudomonads was analyzed with maximum likelihood algorithm and compared with the corresponding sequence of a broad range of *rpo*D sequences from *Pseudomonas* representative type strains available in Genbank. This placed the Moroccan isolates in two separate well supported clusters suggesting that these lineages are genotypically heterogeneous and might belong to a new species within *Pseudomonas sensu stricto*. Comparative phylogenetic analyses with *rpo*D gene sequences was performed to assign the Moroccan isolates with high resolving power to *Pseudomonas* species. The phylogenetic interrelatedness among *Pseudomonas* taxa inferred by *rpo*D gene-based phylogeny was congruent with that inferred by other phylogenies. Our robust maximum likelihood phylogenetic analysis permitted fine differentiation of these *Pseudomonas* isolates, suggesting our *rpo*D gene-based phylogeny is valuable for fine differentiation and efficient classification of closely related *Pseudomonas* isolates, as it was found in other hallmark studies, especially for resolving closely related isolates in microbial ecology^40,41^. This study highlights also the necessity for complete genomic sequencing of these isolates for strong phylogenomic taxonomy to facilitate robust assignment of these species and to avoid potential taxonomic inconsistencies.

This study showed that the new *Pseudomonas* sp. isolates are able to produce indole-3-acetic acid, ammonia, and siderophores and solubilize phosphate.

Phosphate solubilizing is explained by the production of various organic acids and enzymes^42,43^. These factors transform insoluble phosphates into substances that can be easily assimilated by plants^44–46^. Among these organic acids are gluconic, tartaric, and oxalic acids^42,43,47^. Gluconic acid is the main component during solubilization^48,49^ Indole-3-acetic acid^50^ is the most important auxin produced by bacteria, plants and fungi. I-3-AA initiates root, leaf and flower development^51^. Its importance lies in its central role in cell division, elongation, fruit development and senescence^52^. Siderophores themselves are useful for phytostabilization. They facilitate plant growth and coalescence of metals and reduce metal bioavailability in soil^53^; as such they are of use in bioremediation of soils. These characteristics determine plant growth in general. As a result, the five novel isolates of *Pseudomonas* selected can be considered as a new PGPR that have an important role as biological fertilizers with bioremediation qualities^6^. *Pseudomonas* enhances nutrient bioavailability and bioassimilation. The use of these bacterial complements may lead to reduced application rates or even elimination of chemical fertilizers^52^. PGPR increase chlorophyll formation in plant leaves, which enhances photosynthesis^54^. Photosynthesis is the primary metabolic process by which plants grow^55^. PGPR affect healthy plant growth and have a pesticidal role in protecting plants from soil pathogens.

Such diverse benefits have made PGPR a potential resource in themselves. Further work should use this new species as a new candidate for biofertilizers on other plants, aiming at preventing damage to ecosystem structure since “the tapestry becomes threadbare and begins to fall into tatters, becoming irreparable even for the most capable weaver, the last remnants serving essentially no function whatsoever, without all the fibers in their proper places, without all the right words, without all the integral elements of the fabric of life”^56^.

### Future investigation is recommended with both biochemical and molecular approaches

We should elucidate the pathways of the mechanisms involved in this approach such as auxins^57^, siderophores and phosphate solubilization^58^. In addition, we suggest that future studies make use of Bayesian approaches to unify the different subjective areas to form a predictive function for the effectiveness and application of PGPR mechanisms in alternative crop species.

## Methods

To achieve our objectives, we followed the protocols shown in sections 4.1–4.9.

### Isolation of *Pseudomonas* spp

The samples were collected from an experimental farm of the National Institute for Agricultural Research, Agadir, southwestern Morocco (30°02′42.2“N 9°33′13.4“W) in 2016 from tomato roots and rhizospheric soil. For each sample, 500 g was collected from healthy tomato plants in a greenhouse. Samples were kept in the laboratory at 4 °C before analysis.

The bacterial communities of the rhizosphere (RS), the rhizoplane (RH) and the endorhizosphere (ER) were isolated as defined by Dommergues and Mangenot^59^ through the following steps:

(i). To isolate bacteria from the rhizosphere, the roots were carefully shaken. Then, 1 g of rhizospheric soil was added to 9 ml of sterile physiological water, and the mixture was agitated at 120 rpm for 2 min.
(ii). To isolate bacteria from the rhizoplane, the rhizospheric soil was dislodged from the fresh roots, and 1 g of root segments was agitated for 2 hours in 9 ml of sterile physiological water at 120 rpm.
(iii). To isolate bacteria from the endorhizosphere, the surface of root segments was subsequently disinfected using 2.5% sodium hypochlorite solution for 3 min^60^, rinsed three times with sterile distilled water and 1 g was blended in 9 ml of sterile physiologic water.
(iv). Serial dilutions were separately prepared from the extracts (RS, RH and ER), and 0.1 ml of each dilution was seeded onto King B medium to isolate and quantify fluorescent *Pseudomonas* spp.^61^. Three replicates were made for each extract, and the plates were incubated at 28 °C for 48 hours. Fluorescent colonies on King B medium under UV light were subcultured twice before storage at 4 °C on yeast dextrose carbonate (YDC) agar and at −80 °C in 40% glycerol^62,63^.

### Greenhouse seed germination and seedling height measurement

Tomato seeds (Campbell 33) were disinfected with 2.5% sodium hypochlorite solution for 3 min57, rinsed three times with sterile distilled water and air-dried. These seeds were inoculated by soaking for 30 min in a suspension of bacteria (10^8^cfu/ml) amended with 2% carboxymethylcellulose (CMC) and air-dried for 12 hours. The treated seeds were sown in disinfected cell trays (two seeds per cell) containing a mixture of sterilized sand and peat (1:2 v/v). Controls for the experiment were treated with a mixture of sterile distilled water and 2% CMC. The trays were placed in an experimental greenhouse set with a photoperiod of 12 hours, a temperature of 25 ± 2 °C, and a relative humidity (RH) of 80 ± 5% and were watered every 2 days. The rate of germination compared to that of the control was determined five days after sowing. Seedling height measurements were made 20 days after germination. Three replicates were carried out for all treatments with 10 seeds.

### Bacterial characterization

The five selected bacteria were characterized for the following biochemical traits: Gram test, motility, oxidase, catalase, glucose fermentation, arginine-dihydrolase, nitrate reduction, gelatin hydrolysis, levan production, manitol utilization, and carbon source utilization^14,18,64,65^.

Bacterial isolates were cultured for 24 hours, and DNA was extracted using a Bioline ISOLATE II Genomic DNA Kit (Bioline, Australia) following the manufacturer’s guidelines for cell culture extractions with the following adaptation: three to four colonies from fresh culture were suspended in sterile water as the starting material. Extracted DNA was visualized using gel electrophoresis on a 1% agarose gel prestained with ethidium bromide electrophoresed at 100 V for 40–60 min and viewed under UV light. DNA was stored at −20 °C for further use.

#### Molecular detection and partial rpoD sequencing

A fragment of the *rpo*D gene was PCR-amplified with the primers Ps*rpo*D FNP1 (5′-TGAAGGCGARATCGAAATCGCCAA-3′) and Ps*rpo*Dnprpcr1 (5′-YGCMGWCAGCTTYTGCTGGCA-3′)^66^. PCR was performed in a 25 µl volume containing 1 µlof DNA (25 ng/µl), 2.5 µlof 10 × PCR buffer with 20 mM MgCl_2_ (Roche), 2.5 µlof dNTPs (2 mM each), 0.75 µlof forward primer (10 μM), 1.5 µlof reverse primer (10 μM) and 0.125 µlof FastStart polymerase (5 U/µl; Roche). The PCR temperature profile was performed according to Parkinson *et al*.^66^. All amplification products were purified with the GeneJET PCR Purification Kit (Thermo Fisher Scientific Inc.) and sequenced with both forward and reverse primers using a commercial service (Macrogen Inc.). The *rpo*D amplicons were trimmed to 578 nucleotides according to Parkinson *et al*.^66^ with BioNumerics 7.1 software (Applied Maths, Belgium). The 578 nt sequence was used to classify the bacterial isolates at the species level by a BlastN query against the nucleotide database of NCBI. All *Pseudomonas* strains and isolates used in this study for sequencing and phylogenetic analysis are detailed in supplement material (Table S1).

#### rpoD sequences and phylogenetic analysis

The obtained Sanger sequences from the *rpoD* genomic region were assembled, analyzed and aligned using the BioNumerics 7 (Applied Math version 7.6.1). Sequence identity of the Moroccan *Pseudomonas* isolates was confirmed by similarity search using the BLASTn program in GenBank (https://blast.ncbi.nlm.nih.gov/Blast.cgi). In addition to Moroccan *rpo*D partial sequences, a selection of nucleotide sequences of the *rpo*D all representative *Pseudomonas* isolates from different countries and hosts were retrieved from GenBank, aligned and used for phylogenetic analyses and molecular evolutionary genetics analysis. The evolutionary history and phylogenetic tree was inferred by using the Maximum Likelihood method and Tamura-Nei model^67^. The percentage of trees in which the associated taxa clustered together is shown next to the branches. Initial tree(s) for the heuristic search were obtained automatically by applying Neighbor-Join and BioNJ algorithms to a matrix of pairwise distances estimated using the Maximum Composite Likelihood (MCL) approach, and then selecting the topology with superior log likelihood value. The robustness of the internal branches was tested with assessment of the confidence of branching patterns by bootstrap analysis with 500 pseudo-random iterations. The tree was drawn to scale, with branch lengths measured in the number of substitutions per site. This analysis involved 117 nucleotide sequences. Codon positions included were 1st + 2nd + 3rd + Noncoding. There were a total of 584 positions in the final dataset. Evolutionary analyses were conducted in MEGA X^68^.

### Plant growth assay

Testing the effects of the five *Pseudomonas* isolates on tomato plant growth, substrate that was a mixture of sand and peat (1:2 v/v) was inoculated by strain suspensions (10^8^cfu/g)^34^ and was distributed in plastic pots (4 l). Twenty days after sowing, 10 seedlings of the same height that grew out from inoculated seeds were transplanted to the pre-prepared substrate. Plants were grown under typical greenhouse growing conditions (min and max temperatures:18°Cand 26 °C, respectively, 75 ± 5%relative humidity)^69^ and were watered every 2 days and fertilized each week with a commercial nutrient solution (NPK 20-20-20)^34^. The control plants grown from untreated seeds were transplanted into soil substrate without inoculants. All treatments were replicated three times. The pots containing plants were arranged in a completely randomized design. After 20 days, the plant length (aerial region), collar diameter and number of leaves were measured.

### Phosphate solubilization

Phosphate solubilization by the isolated fluorescent *Pseudomonas* isolates was tested in solid media by the method described by Nautiyal^70^. Ten microliters of the culture of each strain was spotted on the surface of the solid media in Petri dishes. The solubilization capacity was assessed by the transparent area formed around the colony. Ten days after incubation at 30 °C, the diameter of the solubilization halo (DSH) was determined by the following formula (Eq. 1):

1 $$DSH=THD-CD$$

where THD is the total halo diameter, and CD is the colony diameter.

Quantitative estimation of phosphate solubilization in broth was carried out in Erlenmeyer flasks (250 ml) containing 100 ml of NBRIP medium^70^. The flasks were incubated at 28 °C for 5 days at 120 rpm. Then, 5 ml of each isolate culture was centrifuged at 3000 rpm for 20 min. Phosphate concentration in culture supernatant was estimated as described by Olsen and Sommers^71^. Three replicates were used, and a standard calibration curve was made with KH_2_PO_4_ solution (Sigma-Aldrich).

### Indole-3-Acetic acid production

I-3-AA production was detected as described by Bric *et al*.^72^. Cultures were maintained on Luria-Bertani (LB) agar medium (Sigma-Aldrich Chemical Co., St. Louis, MO). The pH was adjusted to 7.5 before autoclaving. The LB medium was supplemented with 5 mM L-tryptophan. Agar plates (9-cm diameter) were inoculated using sterile toothpicks; each inoculated Petri dish was overlaid with an 82-mm diameter disk of Whatman paper (No.2). The plates were transferred into an incubator at 27 °C for 3 days. Once the diameter of colonies on LB medium was 2 mm, I-3-AA was present. Whatman disks were treated with Salkowski reagent (2% 0. 5 MFeCl_3_ in 35% perchloric acid). Tests were carried out at room temperature. Bacteria producing I-3-AA were identified by the formation of a characteristic red halo. Three replicates were performed for each isolate.

The production of I-3-AA by the five fluorescent *Pseudomonas* isolates was evaluated spectrophotometrically, with the presence being identified at 535 nm. Liquid cultures were prepared in 250-ml flasks containing 100 ml of 50% TSB, with 200 mg/ml L-tryptophan (Sigma-Aldrich). The flasks were inoculated with 100 µl of each isolate culture (10^8^cfu/ml) and incubated overnight (approx. 12 hours) at 27 °C^23^. Following inoculation, we observed an incubation period of 72 hours on a rotary shaker (150 rev/min, 28 °C). Bacterial cells were removed by centrifugation (4000 g, 10 min). One milliliter of each supernatant was mixed vigorously with 2 ml of Salkowski reagent. The mixture was incubated at room temperature (24 °C) for 20 min. I-3-AA production was observed as a pink-red color, and the absorbance was measured at 535 nm using an Optizen 3220UV Double Beam UV-Vis spectrophotometer (Mecasys, Korea). The concentration of I-3-AA was determined using a standard curve prepared using serial dilutions of a 50 mg/ml I-3-AA (Sigma-Aldrich) solution in 50% TSB. Four replicates were used for each treatment^23^.

### Root colonization

The following steps were carried out: (i) spontaneous antibiotic-resistant mutants were obtained by transferring bacteria to King B medium containing rifampicin (180 µg/ml) (Sigma-Aldrich)^73^. To prevent contamination by fungi, cycloheximide (100 µg/ml) (Sigma-Aldrich) was added to the medium. Colonies growing in this medium were serially transferred three times to the same medium and stored at 4 °C; (ii) tomato seeds (Campbell 33) were disinfected, treated with rifampicin-resistant bacteria and sown in a mixture of sand and peat (1:2, v/v); (iii) After 2 weeks, plants and the soil adhering to the roots were carefully removed. Then, roots were placed on King B medium supplemented with rifampicin (180 µg/ml) and cycloheximide (100 µg/ml). The plates were incubated at 28 °C for 48 hours, and root colonization was evaluated on the basis of the growth of the introduced fluorescent *Pseudomonas* along the roots^34^.

### Production of siderophores

Siderophore production was tested qualitatively using Chrome Azurol S medium (CAS-medium)^74^. Each fluorescent *Pseudomonas* isolate was streaked on the surface of CAS agar medium and incubated at 28 °C for 3 days. Siderophore production was confirmed by observation of an orange halo around the colonies after incubation. Three replicates were performed.

Quantitative analysis of siderophore production was performed in King B liquid medium inoculated with 100 µl of *Pseudomonas* isolate culture (10^8^cfu/ml) and incubated at 28 °C for 72 hours. Cultures were centrifuged at 5000 rpm for 30 min, and 500 µl of the supernatant was mixed with 500 µlof CAS solution. The color changed from blue to orange, indicating siderophore production. After 20 min of incubation, optical density was measured by an Optizen 3220UV Double Beam UV-Vis spectrophotometer (Mecasys, Korea) at 630 nm. The percentage of siderophores was calculated using the following formula (Eq. 2):

2 $${\rm{ \% }}\,Siderophores=\frac{RA-SA}{RA}\ast 100$$

where RA represents the absorbance of the reference (CAS reagent), and SA represents the absorbance of the sample^75^.

### Ammonia production

Ammonia production was measured by growing cultures in peptone water with Nessler’s reagent^76^. A color change from brown to yellow indicates ammonia production; optical density was measured using an Optizen 3220UV Double Beam UV-Vis spectrophotometer at 450 nm^77^. The concentration of ammonia was estimated based on a standard curve of ammonium sulfate (Sigma-Aldrich) ranging from 0.1 to 1 µmol/ml. Sterile peptone water served as a negative control.

### Data analysis

Data were subjected to ANOVA. Data for plant growth experiments are presented as the means ± standard deviation. Any difference mentioned is significant at p < 0.01 using the Newman–Keuls test^78^ (details are provided in a statistics supplementary file).

## Supplementary information

Supplementary info and Datasets
