# Supplementary material for: Applications of New Rhizobacteria Pseudomonas Isolates in Agroecology via Fundamental Processes Complementing Plant Growth
Source: Sci Rep. 2019 Sep 6;9:12832. doi: 10.1038/s41598-019-49216-8 (PMC6731270; doi:10.1038/s41598-019-49216-8)
Supplement: Supplementary file 1 — Supplementary info and Datasets [file 41598_2019_49216_MOESM1_ESM.pdf]

Supplement files

Applications of New Rhizobacteria *Pseudomonas* Isolates in Agroecology via Fundamental Processes Complementing Plant Growth

[1,2]Qessaoui R., [1]R. Bouharroud, [3]J. N. Furze, [1]M. El Aalaoui, [1]H. Akroud, [1]A. Amarraque, [4]R. Tahzima, [4]J. Van Vaerenbergh, [2-3]E H. Mayad, [2]B. Chebli

[1]Research Unit of Integrated Crop Production, Centre Regional de la Recherche Agronomique d'Agadir, Morocco

[2]Biotechnology and Environmental Engineering Team, Laboratory for Process Environmental and Energy Engineering, National School of Applied Sciences, Ibn Zohr University, PO Box: 1136/S, Agadir, Morocco.

[3]Laboratory of Biotechnologies and Valorization of Natural Resources Faculty of Sciences - Agadir, Ibn Zohr University, Agadir, Morocco

[4]Unit Plant – Crop Protection, Institute for Agricultural and Fisheries Research, Burgemeester van Gansberghelaan 96, BE-9820 Merelbeke, Belgium

Corresponding author: bouharroud@yahoo.fr, Phone: +212662062484

Supplementary datasets

Biostatistics is a branch of applied statistics (Mathematics) to a wide range of topics in biology. It must be taught with the focus being on its various applications in scientist research. The utility of agricultural statistics is even more important. The quantitative agricultural researches, in fact, are largely based on statistical data.

In this study we have measured plant growth parameters like seeds germination, seedling height, the length of plants, collar diameter and the number of leaves in addition to mechanisms parameters such as the ability to solubilize phosphate, produce siderophore, ammonia, indole-3-acetic acid and colonize the roots of tomato plants.

To scientifically value these measurements, data analysis were subjected to one-way analyses of variance (ANOVA) using the following equations

$$F_{cal} = \frac{MSF}{MSE} \qquad \text{Eq1}$$

Where MSF is the mean square of treatments and MSE is the mean square of error.

The one-way ANOVA tests the null hypothesis (H_0) and compares the means between the groups and determines the difference if it exists,

$$H_0 : x_1 = x_2 = x_3 = \cdots = x_n$$

Where *x*: Group mean and *n*: number of groups. If, however, H_0 is rejected, the Alternative hypothesis (H_A) is accepted

$$H_A : x_1 \neq x_2 \neq x_3 \neq \cdots \neq x_n$$

Alternative hypothesis (H_A) means that there are at least two group means that are statistically significantly different from each other. In this of significant effects, comparisons of differences between groups (*Pseudomonas* strains and the control) were performed using Newman-keuls test, which is a stepwise test based on q-statistic, which compares every mean with every other mean in a pair-wise fashion using the following equation.

$$q = \frac{\bar{X}_A - \bar{X}_B}{\sqrt{\frac{MSE}{n}}} \qquad \text{Eq 2}$$

Where q is the Studentized Range $\bar{X}_A$ and $\bar{X}_B$ are the group means being compared, and n is the number of observations per group. The calculated q statistic is compared to the critical values listed in the studentized range. Then, we will deduce the degree of difference between the tested groups. Any difference mentioned is significant at p=0.01.

Example of calculation;

To clarify the steps and equations were used, the plants height parameter was chosen as an example.

The data below resulted from measuring the plants height treated by six different treatments (including control). The treatments were replicated 10 times.

Table 1 A set of data to illustrate the Newman-Keuls tests

Experimental Group					
**Control**	Q6B	Q1B	Q7B	Q14B	Q13B
32	34	34	36,5	33	39
32	38	37	35	36	40
35	34	41	42	39	44,5
32	36	39	38	33	40
24	39	36	38	49	44,5
31	37	38	36	36	38

	31	35	35	35	43	37
	30	35,5	37	34	42	41
	30	35,5	40	40	38	41
	31	38	38	36	40	40
Total (T)	308	362	375	370,5	389	405
Mean ($\bar{X}_i$)	30,8	36,2	37,5	37,05	38,9	40,5
N=60,n=10						

1- Compute CM, the correction for the mean.

$$CM = \frac{(\sum_{i=1}^{6}\sum_{j=1}^{10} Xij)^2}{N_{total}} \qquad \text{Eq 6}$$

$$CM = \frac{(\text{Total of all observations})^2}{N_{total}} = \frac{(2209.5)^2}{60} = 81364{,}84$$

2- Compute the total SS (SST).

$$SST = \sum_{i=1}^{6}\sum_{j=1}^{10} Xij^2 - CM \qquad Eq\ 7$$

$$SST = (32^2 + 32^2 + 35^2 + \cdots + 37^2) - CM = 1015{,}41$$

3- Compute SSF, the treatment sum of squares

$$SSF = \sum_{i=1}^{6} \frac{T_i^{\,2}}{n} - CM \qquad Eq\ 8$$

$$SSF = \frac{308^2 + 362^2 + 389^2 + \cdots + 375^2}{10} - 81364{,}84 = 550.085$$

4- Compute SSE, the error sum of squares.

$$SSE = SST - SSF \qquad Eq\ 9$$

$$SSE = 465.325$$

5- Compute MSF and MSE ; MSF is the mean square of treatments and MSE is the mean square of error

$$* MSE = \frac{SSE}{N - Z} \qquad \text{Eq 10}$$

$$MSE = 8.62$$

$$* MSF = \frac{SSF}{n - 1} \qquad \text{Eq 11}$$

$$MSF = \frac{550.085}{10 - 1} = 110{,}02$$

6- Calculus of The F-test using equation 1(Eq 1)

$$F_{cal} = \frac{MSF}{MSE} = \frac{110.02}{8.62} = 12.76$$

Using the F distribution table at α =0. 01, we have $F_{0.\ 01.\ 5,54}$ = 3. 377. $F_{calculated}$ is much larger than the $F_{critical}$, so we reject the null hypothesis (H_0) and conclude that there is a significant difference between the treatment means. But we don't know exactly where the difference exists. In this case, Newman-keuls test has to be used. For this test, the group means are ordered from the smallest to the largest. The test starts by evaluating the largest difference which corresponds to the difference between Q13B and the control: $\bar{X}_6$: $\bar{X}_1$

N is the total number of measures and K is the number of treatments, and a parameter R, which is the number of means being tested. (N=60, K=6, df = 54, MSE = 8,62, n=10, and α=0. 01)

1st - (R=6, $q_{critical}$=4. 99)

$\bar{X}_6$; $\bar{X}_1$

$$q = \frac{\bar{X}_6 - \bar{X}_1}{\sqrt{\frac{MSE}{n}}} = \frac{40{,}5 - 30.8}{\sqrt{\frac{8.62}{10}}} = 10.45$$

The $q_{observed}$ is greater than $q_{critical}$ and H_0 is rejected for the largest pair. That means there is a significant difference between Q13B and the control (α=0. 01)

2nd - Now we proceed to test the means with a range of 5, namely the differences between $\bar{X}_5$: $\bar{X}_1$ and between $\bar{X}_6$:$\bar{X}_2$ with α=0. 01, R = 5 and, $q_{critical\ (r,\ df)}$ = 4. 818

- $\bar{X}_5$: $\bar{X}_1$

$$q = \frac{\bar{X}5 - \bar{X}1}{\sqrt{\frac{MSE}{n}}} = \frac{38.9 - 30.8}{\sqrt{\frac{8.62}{10}}} = 9{,}8$$

The $q_{observed}$ is greater than $q_{critical}$ and H_0 is rejected for this pair. That means there is a significant difference between Q14B and the control (α=0. 01)

- $\bar{X}_6$: $\bar{X}_2$

$$q = \frac{\bar{X}6 - \bar{X}2}{\sqrt{\frac{MSE}{n}}} = \frac{40.6 - 36.2}{\sqrt{\frac{8.62}{10}}} = 4{,}36$$

The $q_{observed}$ is smaller than $q_{critical}$ and H_0 is accepted for this. That means there is not a significant difference between Q13B and Q6B (α=0. 01)

3[rd-] Now we proceed to test the means with a range of 4, namely the differences between $\bar{X}_4$: $\bar{X}_1$, $\bar{X}_5$:$\bar{X}_2$ and between $\bar{X}_6$: $\bar{X}_3$ with α=0. 01, R = 4 and, $q_{critical\ (r,\ df)}$ = 4. 594

- $\bar{X}_4$: $\bar{X}_1$

$$q = \frac{\bar{X}4 - \bar{X}1}{\sqrt{\frac{MSE}{n}}} = \frac{37.5 - 30.8}{\sqrt{\frac{8.62}{10}}} = 7{,}21$$

The $q_{observed}$ is greater than $q_{critical}$ and H_0 is rejected for this pair. That means there is a significant difference between Q1B and the control (α=0. 01)

- $\bar{X}_5$: $\bar{X}_2$

$$q = \frac{\bar{X}5 - \bar{X}2}{\sqrt{\frac{MSE}{n}}} = \frac{38.9 - 36.2}{\sqrt{\frac{8.62}{10}}} = 2{,}91$$

The $q_{observed}$ is smaller than $q_{critical}$ and H_0 is accepted for this. That means there is not a significant difference between Q14B and Q6B (α=0. 01)

- $\bar{X}_6$: $\bar{X}_3$

$$q = \frac{\bar{X}6 - \bar{X}3}{\sqrt{\frac{MSE}{n}}} = \frac{40.5 - 37.05}{\sqrt{\frac{8.62}{10}}} = 3{,}71$$

The $q_{observed}$ is smaller than $q_{critical}$ and H_0 is accepted for this. That means there is not a significant difference between Q13B and the Q7B (α=0. 01)

4th- Now we proceed to test the means with a range of 3, namely the differences between $\bar{X}_3$: $\bar{X}_1$, $\bar{X}_4$:$\bar{X}_2$, $\bar{X}_5$: $\bar{X}_3$ and between $\bar{X}_6$: $\bar{X}_4$ with α=0. 01, R = 3 and, $q_{critical\ (R,\ df)}$ = 4. 282

- $\bar{X}_3$: $\bar{X}_1$

$$q = \frac{\bar{X}3 - \bar{X}1}{\sqrt{\frac{MSE}{n}}} = \frac{37.05 - 30.8}{\sqrt{\frac{8.62}{10}}} = 6.73$$

The $q_{observed}$ is greater than $q_{critical}$ and H_0 is rejected for this pair. That means there is a significant difference between Q7B and the control (α=0. 01)

- $\bar{X}_4$: $\bar{X}_2$

$$q = \frac{\bar{X}4 - \bar{X}2}{\sqrt{\frac{MSE}{n}}} = \frac{37.5 - 36.2}{\sqrt{\frac{8.62}{10}}} = 1{,}4$$

The $q_{observed}$ is smaller than $q_{critical}$ and H_0 is accepted for this. That means there is not a significant difference between Q1B and the Q6B (α=0. 01)

- $\bar{X}_5$: $\bar{X}_3$

$$q = \frac{\bar{X}5 - \bar{X}3}{\sqrt{\frac{MSE}{n}}} = \frac{38.9 - 37.05}{\sqrt{\frac{8.62}{10}}} = 1.99$$

The $q_{observed}$ is smaller than $q_{critical}$ and H_0 is accepted for this. That means there is not a significant difference between Q14B and the Q7B (α=0. 01)

- $\bar{X}_6$: $\bar{X}_4$

$$q = \frac{\bar{X}6 - \bar{X}4}{\sqrt{\frac{MSE}{n}}} = \frac{40.5 - 37.5}{\sqrt{\frac{8.62}{10}}} = 3.23$$

The $q_{observed}$ is smaller than $q_{critical}$ and H_0 is accepted for this. That means there is not a significant difference between Q13B and the Q1B (α=0. 01)

5th- Now we proceed to test the means with a range of 3, namely the differences between $\bar{X}_2$: $\bar{X}_1$, $\bar{X}_3$:$\bar{X}_2$, $\bar{X}_4$: $\bar{X}_3$ and between$\bar{X}_5$: $\bar{X}_4$ with α=0. 01, R = 2 and, $q_{critical\ (R,\ df)}$ = 3. 762

- $\bar{X}_2$: $\bar{X}_1$

$$q = \frac{\bar{X}2 - \bar{X}1}{\sqrt{\frac{MSE}{n}}} = \frac{36.2 - 30.8}{\sqrt{\frac{8.62}{10}}} = 5.82$$

The $q_{observed}$ is greater than $q_{critical}$ and H_0 is rejected for this pair. That means there is a significant difference between Q6B and the control (α=0. 01)

- $\bar{X}_3$: $\bar{X}_2$

$$q = \frac{\bar{X}3 - \bar{X}2}{\sqrt{\frac{MSE}{n}}} = \frac{37.05 - 36.2}{\sqrt{\frac{8.62}{10}}} = 0.91$$

The $q_{observed}$ is smaller than $q_{critical}$ and H_0 is accepted for this. That means there is not a significant difference between Q7B and Q6B (α=0. 01)

- $\bar{X}_4$: $\bar{X}_3$

$$q = \frac{\bar{X}4 - \bar{X}3}{\sqrt{\frac{MSE}{n}}} = \frac{37.5 - 37.05}{\sqrt{\frac{8.62}{10}}} = 0.48$$

The $q_{observed}$ is smaller than $q_{critical}$ and H_0 is accepted for this. That means there is not a significant difference between Q1B and the Q7B (α=0. 01)

- $\bar{X}5 : \bar{X}_4$

$$q = \frac{\bar{X}5 - \bar{X}4}{\sqrt{\frac{MSE}{n}}} = \frac{38.9 - 37.5}{\sqrt{\frac{8.62}{10}}} = 1.50$$

The $q_{observed}$ is smaller than $q_{critical}$ and H_0 is accepted for this. That means there is not a significant difference between Q14B and Q1B (α=0. 01)

- $\bar{X}6 : \bar{X}_5$

$$q = \frac{\bar{X}6 - \bar{X}5}{\sqrt{\frac{MSE}{n}}} = \frac{40.5 - 38.9}{\sqrt{\frac{8.62}{10}}} = 1.72$$

The $q_{observed}$ is smaller than $q_{critical}$ and H_0 is accepted for this. That means there is not a significant difference between Q13B and Q14B (α=0. 01)

The table below represents the total sum-up of the Newman-keuls test analysis.

Table 2 Presentation of the results of the Newman-Keuls test for the data from Table 1.

	Experimental Groups					
	Control	Q6B	Q7B	Q1B	Q14B	Q13B
	M_1=30,8	*M_2=36,2*	*M_3=37,05*	*M_4=37,5*	*M_5=38,9*	*M_6=40,5*
Control	0	5. 4*	6. 25*	6. 70*	8. 10*	9. 70*
Q6B		0	0. 85 ^{ns}	1. 30 ^{ns}	2. 70 ^{ns}	4. 30^{ns}
Q7B			0	0. 450 ^{ns}	1. 85 ^{ns}	3. 45 ^{ns}
Q1B				0	1. 40 ^{ns}	3. 00 ^{ns}
Q14B					0	1. 60 ^{ns}
Q13B						0

*significative difference, ns: non significative difference(p<0. 01)

The summary of data analysis is also showed in following diagram:

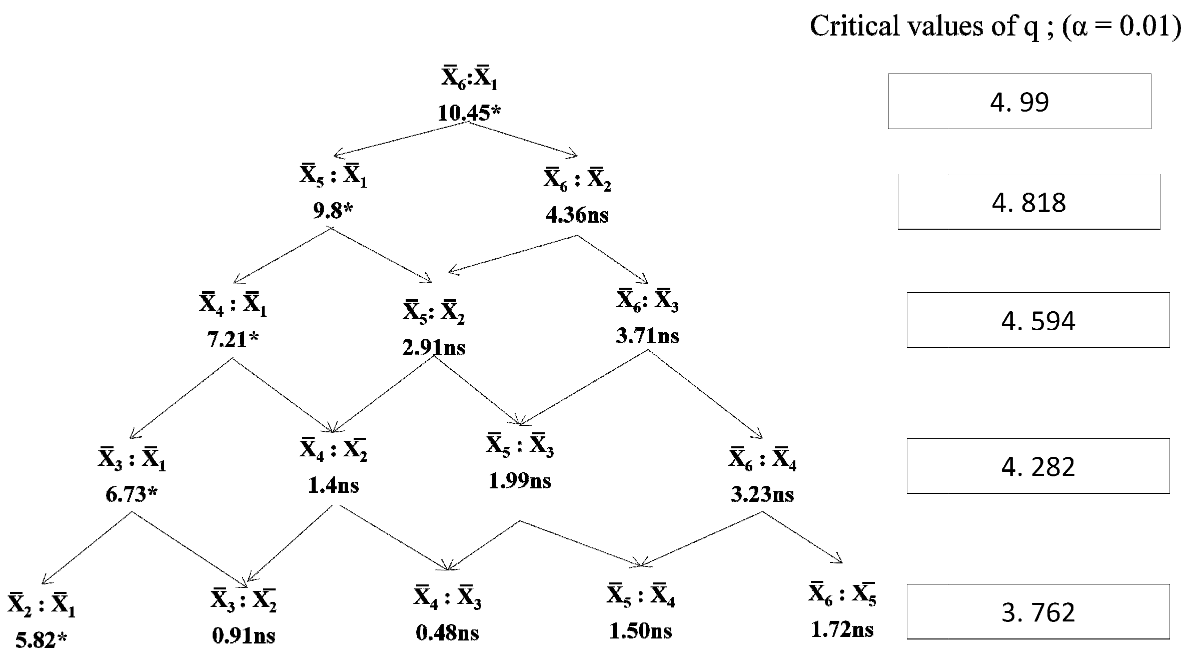

Fig. 1 Newman-Keuls test for the data from plant height. The number below each range is the q observed for that range. at α=0. 01
*significative difference, ns: non significative difference

Statistic analysis of plant length parameter showed that five pseudomonas (Q7B, Q6B, Q1B, Q13B and Q14B), have a difference with control (table 2, fig1) that means that these strains promote significantly the plant height. The longest significant length of plant was obtained with Q13B (fig 1).

Agricultural Statistics have very wide roles, which make them required at different levels, from national to farm level. It is an easy-making for the agricultural policy to take decision and to apply a proot.

Figure S1

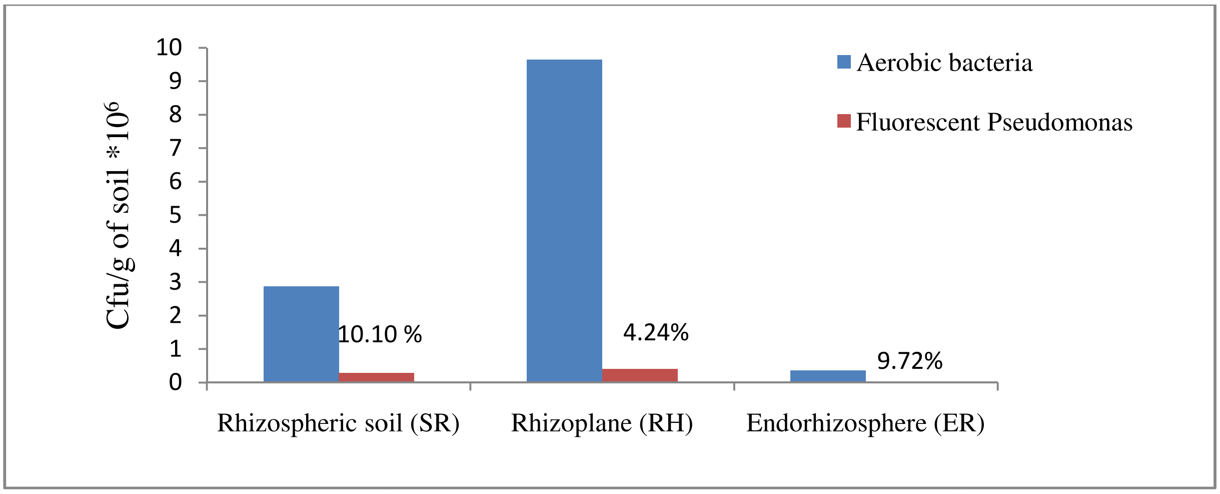

Figure S1 Evaluation of cultivable bacterial flora and fluorescent *Pseudomonas*in the rhizospheric soil (RS),rhizoplane (RH) and endorhizosphere (ER).

Table S1

**Unique ID - CollectionNr**	**OtherNnumbers**	**Genus**	**Species**	**Pathovar**	**Geographic origin**	**Year of isolation**	**Host**	**Corresponding codes**
CFBP 1392T		Pseudomonas	syringae	syringae	United Kingdom	1950		
CFBP 1634PT	LMG 5064, NCPPB 588	Pseudomonas	syringae	garcae	Brazil	1958	Coffea arabica	
CFBP 1674PT	LMG 2330, NCPPB 1898	Pseudomonas	syringae	striafaciens		1958		
CFBP 1908PT	NCPPB 3364	Pseudomonas	syringae	porri	France	1978		
CFBP 2037T	LMG 2210T	Pseudomonas	marginalis		USA	1949		
CFBP 2216PT	LMG 5060, NCPPB 600	Pseudomonas	syringae	coronafaciens	UK	1958		
CFBP 2340PT	LMG 5030, NCPPB 2397	Pseudomonas	syringae	atropurpurea	Japan	1971	Lolium multiflorum	
CFBP 3228PT	LMG 10912, NCPPB 3683	Pseudomonas	syringae	oryzae	Japan	1983		
CFBP 4117PT	NCPPB 3690	Pseudomonas	syringae	oryzae	USA	1983		
CFBP 4850		Pseudomonas	savastanoi	phaseolicola	USA		Pueraria lobata	
CFBP 7286		Pseudomonas	syringae	actinidiae	Italy	2008	Actinidia chinensis	
CIT128		Pseudomonas			Tunisia		Citrus	
CIT40		Pseudomonas			Tunisia		Citrus	
**DSM 14164T**	**LMG 21609T**	**Pseudomonas**	**monteilii**		**France**	**1990**		
DSM 16007T	LMG 24280T	Pseudomonas	moraviensis		Tsjechië	2004		
LMG 1247PT	NCPPB 281, CFBP 1394, PDDCC 3023	Pseudomonas	syringae	syringae	UK	1950	Syringa vulgaris	
LMG 17761T		Pseudomonas	veronii		France	1992		
LMG 17764T		Pseudomonas	rhodesiae		France	1992		
LMG 1794T		Pseudomonas	fluorescens		UK	1951		
LMG 19695T		Pseudomonas	extremorientalis		Russia	1998		
LMG 21318T	Ps 9-14	Pseudomonas	koreensis		Korea	2002		

LMG 21465T		Pseudomonas	poae		Germany	1994		
LMG 2163		Pseudomonas	cichorii		USA	1952		
LMG 21661T		Pseudomonas	graminis		Germany			
LMG 2190T	IFO 3913T	Pseudomonas	synxantha			<1950		
LMG 21995T		Pseudomonas	lurida		Germany	1994		
LMG 2210T		Pseudomonas	marginalis		USA	1949		
LMG 2212	NCPPB 1557	Pseudomonas	marginalis		UK	1963		
LMG 22120T		Pseudomonas	salomonii		France	1976	Allium sativum	
**LMG 2257T**	**IFO 14164**	**Pseudomonas**	**putida**		**USA**	**1973**		
LMG 22709T		Pseudomonas	antarctica		antarctica			
LMG 23068T	DSM 15294T	Pseudomonas	brenneri		France	1997		
LMG 2340		Pseudomonas	sp.		UK	1961		
LMG 2342T	NCPPB 2192T	Pseudomonas	tolaasii		UK	1965		
LMG 28180T		Pseudomonas	simiae		Spain	2003		
LMG 5071PT	NCPPB 2039	Pseudomonas	syringae	maculicola	New Zealand	1965	Brassica oleracea Botrytis group	
LMG 5100		Pseudomonas	viridiflava		USA	1964	Brassica oleracea	
LMG 5398		Pseudomonas	viridiflava		New Zealand	1983	Pisum sativum	
LMG 5496	NCPPB 2786	Pseudomonas	syringae		Greece	1962	Citrus sinensis	
LMG 5507		Pseudomonas	syringae	tomato	Canada	1944	Solanum lycopersicum	
LMG 5830		Pseudomonas	fluorescens		UK	1952		
LMG 5848		Pseudomonas	fluorescens		UK	1980		
LMG 5849		Pseudomonas	fluorescens		UK	1981		

**LMG 5850**		**Pseudomonas**	**sp.**		USA	1960		
LMG 6480		Pseudomonas	viridiflava		Belgium	1984		
LMG 6821		Pseudomonas	fluorescens		Belgium	1984		
LMG2349PT		Pseudomonas	syringae	ulmi	Yugoslavia	1958		
LMG5092PT	NCPPB 2598PT	Pseudomonas	syringae	theae	Japan	1970		
**MN091814**		**Pseudomonas**	**sp.**		**Morocco**	**2018**	**Solanum lycopersicum**	**Q14B**
**MN091815**		**Pseudomonas**	**sp.**		**Morocco**	**2018**	**Solanum lycopersicum**	**Q13B**
**MN091816**		**Pseudomonas**	**sp.**		**Morocco**	**2018**	**Solanum lycopersicum**	**Q12B**
**MN091817**		**Pseudomonas**	**sp.**		**Morocco**	**2018**	**Solanum lycopersicum**	**Q7B**
**MN091818**		**Pseudomonas**	**sp.**		**Morocco**	**2018**	**Solanum lycopersicum**	**Q11B**
**MN091819**		**Pseudomonas**	**sp.**		**Morocco**	**2018**	**Solanum lycopersicum**	**Q9B**
**MN091820**		**Pseudomonas**	**sp.**		**Morocco**	**2018**	**Solanum lycopersicum**	**Q10B**
**MN091821**		**Pseudomonas**	**sp.**		**Morocco**	**2018**	**Solanum lycopersicum**	**Q1B**
**MN091822**		**Pseudomonas**	**sp.**		**Morocco**	**2018**	**Solanum lycopersicum**	**Q4B**
**MN091823**		**Pseudomonas**	**sp.**		**Morocco**	**2018**	**Solanum lycopersicum**	**Q6B**
**MN091824**		**Pseudomonas**	**sp.**		**Morocco**	**2018**	**Solanum lycopersicum**	**Q15B**
NCPPB 1034	LMG 5074	Pseudomonas	syringae	mori	Hungary	1961	Morus alba	
NCPPB 1106		Pseudomonas	syringae	tomato	UK	1961	Solanum lycopersicum	
NCPPB 133		Pseudomonas	syringae	primulae	USA	1939	Primula sp.	
NCPPB 1387		Pseudomonas	syringae	passiflorae	New Zealand	1963		

NCPPB 1437T	LMG 5096	Pseudomonas	cannabina		Hungary	1960	Cannabis sativa	
NCPPB 1626		Pseudomonas	syringae	apii	USA	1964	Apium graveolens var. dulce	
NCPPB 1817		Pseudomonas	syringae	antirrhini	UK	1966	Antirrhinum majus	
NCPPB 1873T	LMG 2152	Pseudomonas	caricapapayae		Brazil	1966		
NCPPB 1879		Pseudomonas	syringae	delphinii	New Zealand	1966	Delphinium sp.	
NCPPB 1921		Pseudomonas	syringae	viburni	USA	1966	Viburnum sp.	
NCPPB 2411PT		Pseudomonas	savastanoi	glycinea	Zimbabwe	1962	Glycine javanica	
NCPPB 247	LMG 1243, LMG 5041, CFBP 4031	Pseudomonas	marginalis		USA	1924	Lactuca sativa	
NCPPB 2488		Pseudomonas	syringae	tagetis	Zimbabwe	1972	Tagetes erecta	
NCPPB 2585PT	LMG 5079, CFBP 2105	Pseudomonas	syringae	pisi	New Zealand	1974	Pisum sativum	
NCPPB 2607T	LMG 13184, LMG 2123	Pseudomonas	amygdali		Greece	1974	Prunus amygdala	
NCPPB 2640		Pseudomonas	syringae	helianthi	Mexico	1974	Helianthus annuus	
NCPPB 2724		Pseudomonas	syringae	berberidis	New Zealand	1975	Berberis sp.	
NCPPB 2848		Pseudomonas	syringae	papulans	Canada	1975	Malus sylvestris	
NCPPB 2995		Pseudomonas	syringae	morsprunorum		1977	Prunus domestica	
NCPPB 3033T	LMG 2220T	Pseudomonas	meliae		Japan	1978	Melia azedarach	
NCPPB 3093		Pseudomonas	syringae	japonica	Japan	1979		
NCPPB 3143		Pseudomonas	syringae	myriceae	Japan	1981	Myrica rubra	

NCPPB 3257PT		Pseudomonas	syringae	philadelphi	UK	1983	Philadelphus coronarius	
NCPPB 3465T	LMG 21627, LMG 22121	Pseudomonas	tremae		Japan	1986	Trema orientalis	
NCPPB 3487T	LMG 21662	Pseudomonas	avellanae		Greece	1987	Corylus avellana	
NCPPB 3681		Pseudomonas	syringae	aesculi	India	1990	Aesculus indica	
NCPPB 3682		Pseudomonas	syringae	hibisci	USA	1990	Hibiscus rosa seinensis	
NCPPB 3686		Pseudomonas	syringae	persicae	New Zealand	1990	Prunus salicina	
NCPPB 3688		Pseudomonas	syringae	photiniae	Japan	1990	Photinia glabra	
NCPPB 3693T	LMG 5694, JCM2400	Pseudomonas	ficuserectae		Japan	1990	Ficus erecta Inubiwa	
NCPPB 3739PT	ICMP 9617	Pseudomonas	syringae	actinidiae	Japan (Shizuoka)	1984	Actinidia chinensis	
NCPPB 3781PT		Pseudomonas	syringae	coriandricola	Germany	1990	Coriandrum sativum var. micocarpur	
NCPPB 3871		Pseudomonas	syringae	actinidiae	Italy	1992	Actinidia chinensis	
NCPPB 52		Pseudomonas	savastanoi	phaseolicola	Canada	1941	Phaseolus vulgarus	
NCPPB 537PT	LMG 5070	Pseudomonas	syringae	lachrymans	USA	1958	Cucumis sativus	
NCPPB 635T	LMG2352, PDDCC 2848	Pseudomonas	viridiflava		Switzerland	1957	Phaseolus sp.	
NCPPB 639T	LMG 2209	Pseudomonas	savastanoi	savastanoi	Yugoslavia	1959	Olea europaea	
NCPPB 871	LMG 5059, CFBP 1617	Pseudomonas	syringae	aptata			Beta vulgaris	
NCPPB 943T	LMG 2162	Pseudomonas	cichorii		Germany	1961		

NCPPB 958		Pseudomonas	syringae	aceris		1961	Acer sp.	
NCPPB 963PT	LMG 2276	Pseudomonas	syringae	ribicola		1961	Ribes aureum	
P16_2014		Pseudomonas	marginalis		Belgium	2014		
P17_2014		Pseudomonas	rhodesiae		Belgium	2014		
P24_2014		Pseudomonas	marginalis		Belgium	2014		
P55		Pseudomonas	syringae	porri	Netherlands	2010		
SB3471/3		Pseudomonas	fluorescens		Bulgaria - Plovdiv	2013		
SB3730		Pseudomonas	sp.		Bulgaria	2014		
SB3731		Pseudomonas	sp.		Bulgaria	2014		
SB3732		Pseudomonas	sp.		Bulgaria	2014		
SB4144/1		Pseudomonas	poae		Bulgaria	2016		
SB4157/1		Pseudomonas	poae		Bulgaria	2016		
SB4158/1		Pseudomonas	fluorescens		Bulgaria	2016		
SB4164/1		Pseudomonas	fluorescens		Bulgaria	2016		
SB4372/2		Pseudomonas	lurida		Bulgaria	2017		
SB4375/1		Pseudomonas	poae		Bulgaria	2017		
SB4389/5		Pseudomonas	lurida		Bulgaria	2017		
SES5		Pseudomonas	rhodesiae					
W5AT15		Pseudomonas	moraviensis					
Y21		Pseudomonas	sp.		Tunisia			
Y58		Pseudomonas	sp.		Tunisia			
